# MAIP1-Related Tumor Immune Infiltration: As a Potential Prognostic Biomarker for Esophageal Cancer

**DOI:** 10.1155/2022/7282842

**Published:** 2022-06-14

**Authors:** Xiaoyu He, Fu Gan, Yongjian Lin, Guoqing Liu, Yunhua Lin, Dongxu Chen

**Affiliations:** ^1^Department of Thoracic Surgery, The Shenzhen Bao'an District Songgang People's Hospital, Shenzhen 518105, China; ^2^Department of Urology Surgery, The Affiliated Hospital of Youjiang Medical University for Nationalities, Baise 533000, China; ^3^Department of Gastrointestinal and Gland Surgery, The First Affiliated Hospital of Guangxi Medical University, Nanning 530021, China; ^4^First Clinical Medical College, Guangxi Medical University, Nanning 530021, China; ^5^Department of Bone and Joint, Nanxishan Hospital of Guangxi Zhuang Autonomous Region, Guilin 541002, China

## Abstract

**Background:**

Esophageal cancer (EC), a common malignant tumor of digestive tract, is also one of the most deadly cancers. Accumulating studies have shown that the initiating and progressing multiple human diseases were closely related to the expression of MAIP. However, the specific roles and mechanisms of MAIP1 in EC remain incompletely defined.

**Purpose:**

This study aims to determine the clinical significance of MAIP1 in EC and explores its potential molecular mechanisms regulating tumor immune infiltration.

**Methods:**

We obtained RNA-seq datasets and corresponding clinical data for EC patients from the Cancer Genome Atlas (TCGA) database via the UCSC Xena browser to extract MAIP1 expression and plot survival curves to determine their prognosis. Based on the differential expression of MAIP1, EC patients were divided into high and low group to investigate the mechanism of MAIP1 in EC. In addition, the single sample gene set enrichment analysis (ssGSEA) quantified the expression of various immune cell signature marker genes and assessed the degree of immune infiltration in EC.

**Results:**

In the TCGA-EC cohort, the overexpression of MAIP1 was observed in tumor tissues compared to normal tissues (*p* = 0.0038). Overall survival analysis showed that EC patients with the overexpression of MAIP1 presented a lower overall survival and worse prognosis (*p* = 0.004). Enrichment analysis revealed that the differential genes (DEGs) between high and low group are involved in biological functions such as extracellular matrix and organization extracellular structure. The results of ssGSEA showed that DCs, iDCs, macrophages, mast cells, and NK cells were significantly different in MAIP1^high^ and MAIP1^low^ groups, and all showed high expression in the MAIP1^low^ group.

**Conclusion:**

We proposed that MAIP1 overexpression was associated with poor prognosis and tumor immune infiltration in EC. At present, there are few MAIP1-related tumor immune infiltration studies in EC, and further investigation is needed.

## 1. Introduction

Esophageal cancer (EC) is one of the most common gastrointestinal tumors, and its global incidence and mortality rates continue to rise [1]. Currently, the standard esophageal cancer treatment strategies include surgery, chemotherapy, radiotherapy, molecular targeted therapy, and combination options [2]. Nevertheless, the 5-year overall survival (OS) rate for patients with EC remains below 15% [1]. An important reason is the lack of effective early diagnosis. The vast majority of patients with EC is usually advanced at the first diagnosis and has a very poor clinical prognosis [3, 4]. Therefore, the current goal of achieving effective treatment remains challenging. It is essential to develop potential biomarkers to improve diagnostic rates and enhance the sensitivity of immunotherapy.

Matrix AAA peptidase-interacting protein 1 (MAIP1) is a protein-coding gene, formerly C2orf47, located on chromosome 2q33.1. The protein encoded by MAIP1 is localized to the mitochondria. König T et al. found that the transit peptide region of the SMDT1/EMRE precursor protein and MAIP1 recognized each other in the mitochondrial matrix, which assures that SMDT1/EMRE is modified by mitochondrial processing peptidases to prevent protein degradation by YME1L1 [5]. Research in MAIP1-related disorders (including spastic ataxia 5 and spinocerebellar ataxia) has shown that the proliferation of 293T cell lines was inhibited by high expression of MAIP1. Furthermore, knockdown of MAIP1 resulted in a significant reduction in apoptosis triggered by sugar and serum starvation. Overall, this gene may have underlying functions in regulating cell proliferation and starvation-induced apoptosis [6]. A recent study likewise suggests that MAIP1 may play an important role in various physiological processes [7]. Therefore, we hypothesized that MAIP1 might be differentially expressed in various tumors and is important in tumorigenesis and progression. Moreover, there were few studies on MAIP1 in EC, especially as a diagnostic biomarker, immunotherapeutic target, and prognosis-related mechanisms.

In this study, we first analyzed the expression of MAIP1 in EC and further estimated the prognostic and diagnostic significance of MAIP1 in EC. Subsequently, we further explored the gene function and related pathways of MAIP1. In conclusion, we identified the relationship between MAIP1 expression and the components of the tumor immune microenvironment and immune checkpoints in EC.

## 2. Materials and Methods

### 2.1. Raw Data Acquisition

The RNA-seq datasets and corresponding clinical data for EC were obtained from the Cancer Genome Atlas (TCGA) database via the UCSC Xena browser (https://xenabrowser.net/). The expression levels of the MAIP1 gene were extracted and screened for clinical characteristics such as survival data, gender, age, and metastasis. The gene expression levels and clinical information were also statistically analyzed.

### 2.2. Survival Analysis

According to the median of MAIP1 expression, EC patients were divided into high and low expression groups, respectively. The R package “survival” was applied to the Kaplan-Meier (KM) survival analysis and survival curve of EC patients. Using the log-rank test, we calculated *p* values and hazard ratios (HR) with 95% confidence intervals (CI) to compare whether the differences in overall survival between groups were statistically significant.

### 2.3. Receiver Operating Characteristic (ROC) Analysis

The ROC plotted by the R package “timeROC” was used to test the diagnostic performance of MAIP1 for EC patients at 1, 3, and 5 years. The AUC value indicated the predictive accuracy of signature of EC, and as the AUC value increases, the predictive power of the gene became higher.

### 2.4. Correlation Analysis of Clinical Traits

The R package “ggpubr” was utilized to analyze the co-expression association between MAIP1 and clinicopathological features (such as gender, pathological stage, and metastasis), and statistical tests were performed. *p* < 0.05 means that the difference is statistically significant.

### 2.5. Difference Analysis between High and Low Expression Groups

Difference analysis of the two subgroups for MAIP1 expression was conducted using the R package “limma.” The criteria for identification of DEGs between groups were a false discovery rate (FDR) adjusted *p* value less than 0.05 and |log2FC| ≥ 1. And use the heat map and volcano map to show the difference in the expression of differential genes between the two groups.

### 2.6. Functional Enrichment Analysis of Differential Genes

Gene ontology (GO) includes cellular component (CC), molecular function (MF), and biological process (BP). Based on the transcriptome, the Kyoto Encyclopedia of Genes and Genomes (KEGG) analysis described the biological signaling pathways that differential genes were mainly involved in. Besides, the R package, “cluster profile” package, can realize differential genes. Pathways of corrected *p* < 0.05 were regarded as significant enrichment.

### 2.7. Gene Set Enrichment Analysis (GSEA)

GSEA analysis of subgroups was performed using GSEA (v 4.0.3) software, and the “c2.cp.kegg.v7.4.symbols.gmt” gene set files were extracted from the molecular signature database (MSigDB, http://www.broadinstitute.org/gsea/msigdb). We used the normalized enrichment score (|NES|) and (FDR) adjusted *p* value as measures of genomic enrichment significance. In addition, the single sample gene set enrichment analysis (ssGSEA) algorithm was performed to quantify immune infiltration.

### 2.8. Immune Cell Infiltration Score

According to the expression of various immune cell characteristic landmark genes, we then assessed the immune-infiltration degree of patients with EC by using the R package “GSVA” in the immune-related gene set files.

### 2.9. Statistical Analysis

All statistical analyses were executed in R software (v.3.6.2). Survival analysis of EC patients based on MAIP1 signature was evaluated using Kaplan-Meier algorithm and ROC curves. Log-rank test was used to validate survival curves. In the Pearson correlation analysis, the correlation coefficient (Cor) > 0.2, *p* < 0.05, was considered correlated. Student *t* test and Welch's *t* test were performed to compare the differences in gene expression and immune infiltration between subgroups. *p* value <0.05 was considered statistically significant.

## 3. Results

### 3.1. Overexpression of MAIP1 Was Potentially Related to EC Pathogenesis

**Figure 1:**
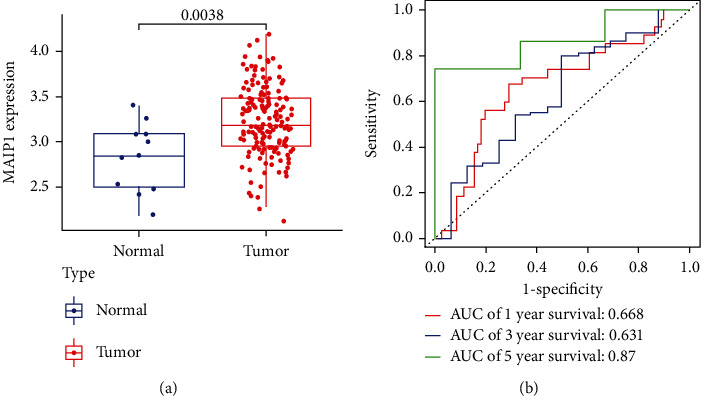
Expression and diagnosis value of MAIP1 in EC. (a) Expression of MAIP1 between EC tissues and normal tissue based on TCGA datasets. (b) Receiver operating characteristic (ROC) curve of EC tissues and normal tissue.

Based on the TCGA database, we identified significantly higher MAIP1 expression in the EC cohort (*p* = 0.0038) (Figure 1(a)). In order to discuss the contribution of MAIP1 signature to EC diagnosis, we fitted the ROC curves.  Figure 1(b) shows that the MAIP1 was highly accurate in differentiating tumor tissue from normal tissue (AUC = 0.668 (1-year survival), AUC = 0.631 (3-year survival), and AUC = 0.87 (5-year survival)). These data strongly suggested that MAIP1 is closely associated with the pathogenesis of EC.

### 3.2. Survival Analysis and Stratification Analysis

**Figure 2:**
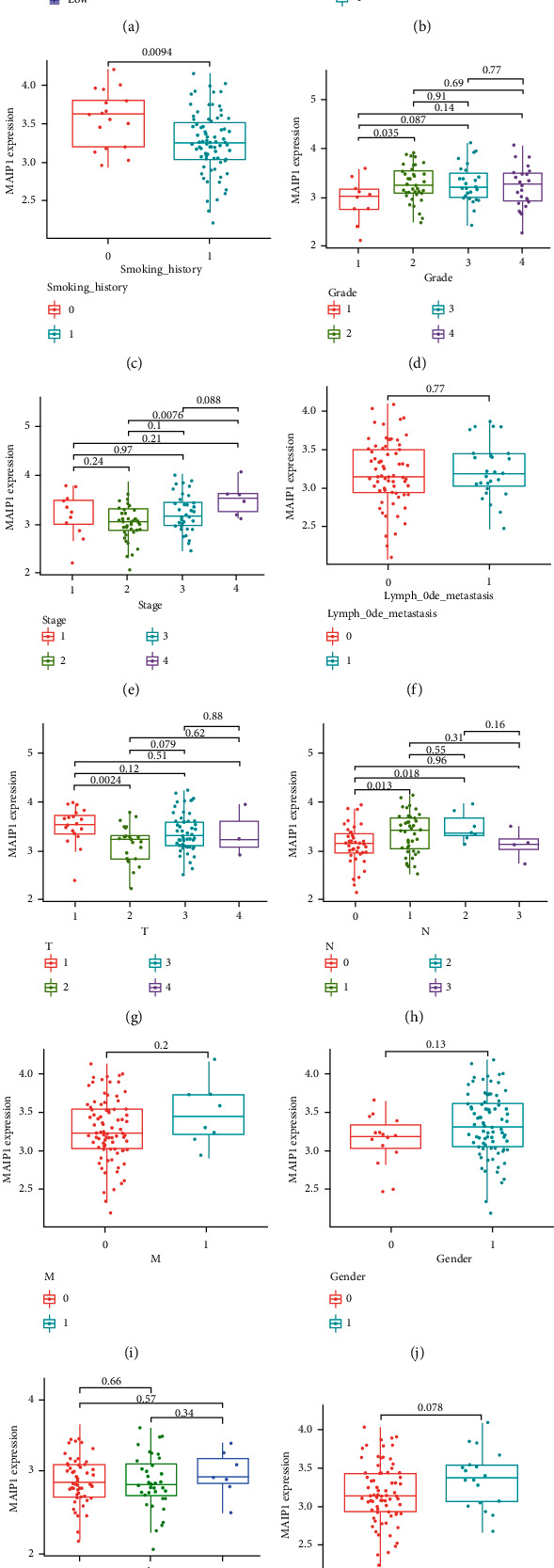
Effect of MAIP1 on prognosis and clinical features in EC. (a) Relationship between MAIP1 expression and patient' OS. (b–l) Expression of MAIP1 in clinical feature values.

By analyzing the difference in overall survival (OS) in MAIP1 subgroups, we found that the overall survival (OS) of patients in the MAIP1 high expression group was significantly lower than that of the MAIP1 low group (*p* = 0.004), demonstrating that the overexpression of MAIP1 was an unfavorable factor affecting the prognosis of EC patients (Figure 2(a)). The level of MAIP1 expression among various clinicopathological characteristics was further investigated by stratified analysis, which revealed pronounced overexpression of MAIP1 in EC patients with alcohol history (*p* < 0.01), smoking history (*p* < 0.01), high tumor grade (*p* < 0.05), advanced clinical stages (*p* < 0.01), and active lymph node metastasis status (*p* < 0.05) (Figures 2(b)–2(l)). Thus, MAIP1 expression is linked to alcohol history, smoking history, cancer stage, tumor development, and the immune microenvironment of metastasis status.

### 3.3. MAIP1 Played Multiple Regulatory Functions in EC

**Figure 3:**
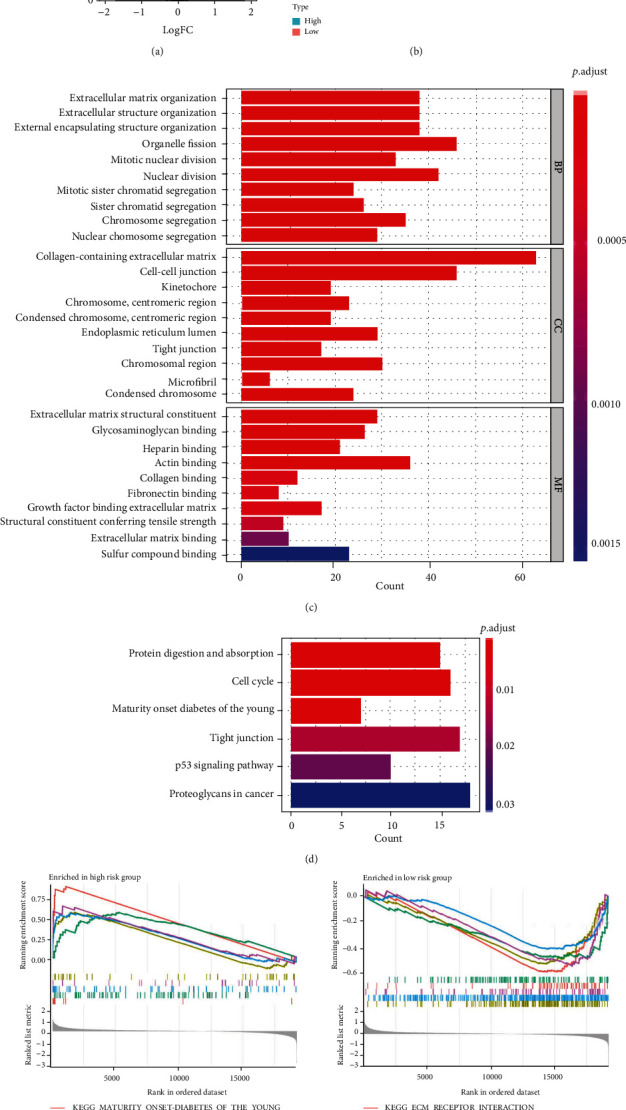
Potential mechanism of MAIP1 in EC. (a) Volcano map of differentially expressed genes between MAIP1^high^ and MAIP1^low^ groups. (b) Correlation heat map of 40 genes with the most obvious positive and negative correlations. (c) Results of GO functional enrichment of DEGs. (d) Results of KEGG functional enrichment of DEGs. (e) GSEA enrichment results for MAIP1^high^ group. (f) GSEA enrichment results for MAIP1^low^ group.

Analysis of the DEGs between MAIP1^high^ and MAIP1^low^ groups in the TCGA-EC cohort revealed 341 upregulated and 286 downregulated DEGs (Figure 3(a)). We selected the 40 genes with the most obvious positive and negative correlations as a correlation heat map (Figure 3(b)). The GO enrichment results showed that the DEGs were involved in biological functions (BP) such as organelle fission, nuclear division, extracellular matrix, and organization extracellular structure; they were also involved in cellular components (CC), such as collagen-containing extracellular matrix, cell−cell junction, and chromosomal region; in addition, they were also involved in molecular functions (MF), including extracellular matrix structural components, collagen binding, and glycosaminoglycan binding, etc. (Figure 3(c)). KEGG analysis showed that the DEGs were mainly enriched in the p53 signaling pathway, cell cycle, protein digestion and absorption, proteoglycans in cancer, and other pathways (Figure 3(d)). GSEA was performed further to understand functional differences between the high- and low-risk groups. High-risk scores were significantly associated with homologous recombination, ribosome, primary immunodeficiency, base excision repair, and proteasome (Figure 3(e)). Meanwhile, low-risk scores were closely associated with valine leucine and isoleucine degradation, citrate cycle, propanoate metabolism, peroxisome, and butanoate metabolism (Figure 3(f)).

### 3.4. MAIP1 Expression Was Associated with Immune Infiltration and Immune Checkpoint Markers

**Figure 4:**
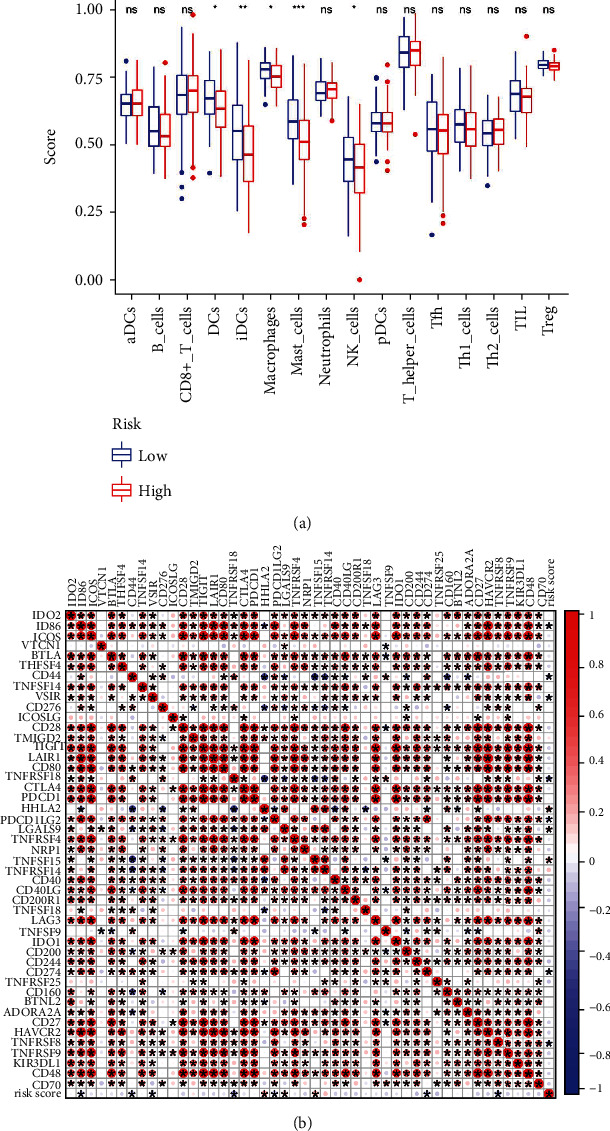
MAIP1 correlated with the immune cell infiltration and immune checkpoint-related genes in EC. (a) Infiltration level of various immune cells in MAIP1^high^ and MAIP1^low^ groups. (b) Profile of correlation between the immune checkpoint-related genes or and risk scores in EC.

**Figure 5:**
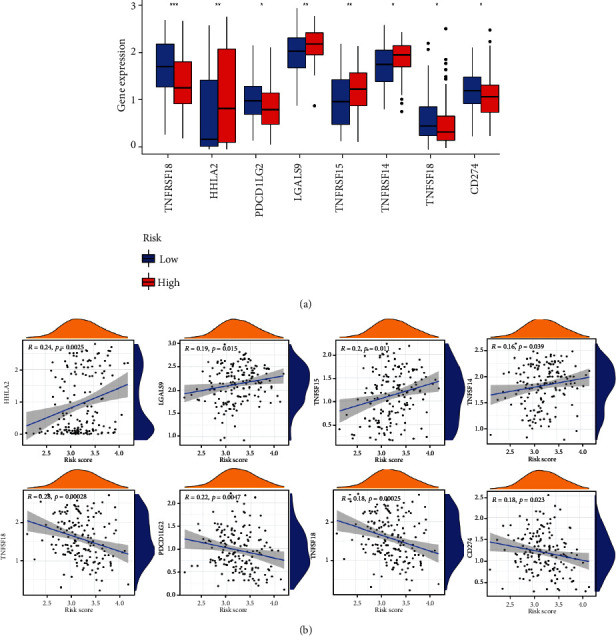
Relationship between MAIP1 and immune checkpoint-related genes. (a) Expression of 8 immune checkpoint-related genes in MAIP1^high^ and MAIP1^low^ groups. (b) Scatter plots show the significant correlation between MAIP1 and the eight immune checkpoint-related genes.

Using the ssGSEA scoring algorithm, the differences of immune cells in the MAIP1 expression groups were studied. It was found that DCs, iDCs, macrophages, mast cells, and NK cells exhibited dramatically different infiltration distributions (Figure 4(a)) and were all highly in the MAIP1^low^ group. We then used correlation analysis to assess the association between MAIP1 and immune checkpoint-related genes, and due to the results, we could reasonably infer that immune function is suppressed in the MAIP1^high^ group (Figure 4(b)). Several key immune checkpoint-related genes, including TNFRSF18, HHLA2, PDCD1LG2, LGALS9, TNFSF15, TNFRSF14, TNFSF18, and CD274, were remarkably different between two groups (Figure 5(a)). Results demonstrated that MAIP1 expression was positively correlated with HHLA2 (*R* = 0.24, *p* = 0.0025), LGALS9 (*R* = 0.19, *p* = 0.015), TNFSF15 (*R* = 0.2, *p* = 0.011), and TNFRSF14 (*R* = 0.16, *p* = 0.039) and negatively correlated with TNFRSF18 (*R* = −0.28, *p* = 0.00028), PDCD1LG2 (*R* = −0.22, *p* = 0.0047), TNFSF18 (*R* = −0.28, *p* = 0.00028), and CD274 (*R* = −0.18, *p* = 0.023) (Figure 5(b)).

## 4. Discussion

We first explored the relevant signaling pathways and clinical value of MAIP1 in EC using the TCGA dataset. Overall, this is the first demonstration that EC patients with high MAIP1 expression are predisposed to a state of low immune infiltration and worse prognosis than patients with low MAIP1 expression. The ROC curve analysis illustrated that MAIP1 could accurately distinguish between EC tissues and non-EC tissues with an AUC value of 0.87 for 5-year survival rate. Moreover, higher MAIP1 expression level also appears to be related to higher tumor stage grade, lymph node migration, and progression of EC.

EC is a kind of lethal and aggressive tumors of the gastrointestinal tract, and in many cases, the prognosis remains detrimental [8]. The present findings suggested that MAIP1 may act as a tumor-promoting factor regulating the TME and immune mechanisms in EC and may serve as a diagnostic biomarker and a predictor of poor prognosis in EC. In this paper, we describe the identification of MAIP1 as a potential regulator of tumor immune cell infiltration in EC patients and highlight the association of MAIP1 expression with a poorer prognosis in this disease. Accumulating studies suggest that EC-related prognostic gene MAIP1 is involved in immune regulation and the tumor microenvironment, as well as the cancer-promoting mechanism. Therefore, our results may offer novel strategies for the development of promising biomarkers, sensitive immunotherapy target, and individualized medicine.

We screened the DEGs for significant different expression of MAIP1 in both high and low groups. To further analyze the possible regulatory mechanisms, functional enrichment analysis of DEGs was performed. Our results showed that these differential genes are involved in biological functions such as extracellular matrix and organization extracellular structure. The KEGG results also indicated that these differential genes are involved in proteoglycans in cancer. The extracellular matrix (ECM) is involved in constituting an essential part of the TME and consists mainly of proteins and glycosaminoglycans [9, 10]. I-type collagen, the most abundant constituent of the ECM, is produced by tumor-associated fibroblasts or cancer-associated fibroblasts and acts on the spaces between cancer cells to enhance tumor stiffness [11]. Dense ECM inhibits diffusion, penetration, and transport of therapeutic drugs. Furthermore, glycoproteins are involved in intercellular adhesion and can be altered to promote the migration of cancer cells [12]. For instance, loss of E-cadherin maintains cell-cell adhesion and communication, which is related to increased invasiveness of cancer cells [13]. The above results suggest that MAIP1 may promote EC development by affecting TME.

A recent reported that the spatial distribution of dendritic cells and macrophages could affect the outcome of esophageal squamous cell carcinoma (ESCC) patients receiving combined chemoradiotherapy or immune checkpoint blockade [14]. Our study showed that MAIP1 was significantly negatively correlated with DCs, iDCs, macrophages, mast cells, and NK cells in EC. In general, MAIP1 overexpression leads to an immunoreactive microenvironment by recruiting immune cells, and such aberrant infiltration contributes to cancer cell proliferation, distant metastasis, and worsens prognosis. M2 macrophages possess a function in mediating immune escape of ESCC cells. When EV-IL-32 derived from ESCC cell lines is internalized by macrophages, it could polarize M2 macrophages via the FAK-STAT3 pathway, thus promoting ESCC evasion from immune surveillance [15]. Li et al. investigated the effect of tumor-associated macrophages on EC and found that the density of M2 macrophage results in abnormal survival and progression of TNM stage in ESCC [16]. Studies have shown that the hyperinfiltration of M2 macrophage produces an adverse response to neoadjuvant therapy for ESCC patients and progresses malignantly in EC [17]. Furthermore, the MC counts are a double-edged sword of tumor immunity, resisting cancer cell proliferation and infiltration during normal counts. In contrast, abnormal counting not only exert the chain responding of tumor angiogenesis, but also is capable of producing multiple cytokines and enzymes involved in the progression and spread of ESCC when excessive [18]. A study showed that mast cells in degranulated form were present in a large amount of paraneoplastic tissue, which increased the possibility of mast cell proliferation causing death due to anaphylaxis in ESCC [19]. The pathological function of mast cells in the EC microenvironment may reveal a novel immune escape system for cancer progression. Previously reported that during endothelial-like differentiation of immature dendritic cells (iDCs), EC tissue could prevent them from differentiating into mature DCs and thus losing their antigen-presenting ability. The response of CTLs against tumors is a heterogeneous prognostic key crosstalk that advances important tumor development in concert with DCs. The activity and function of natural killer cells are restricted by the STAT3 signaling pathway mediated by EC cell-secreted IL-6 and IL-8, which results in the malignancy of ESCC [20]. In the current study, MAIP1 inhibited the infiltration of macrophages and mast cells but also DC cells, iDC cells, and NK cells. Meanwhile, the physiological state of immune cell infiltration could antagonize the above results. This means immune cells infiltration can simultaneously promote and inhibit tumor development. We speculate that the tumor-promoting effect of MAIP1 may be the result of the above immune cell interactions. Our study further analyzed that unusual immune cell infiltration may be potential factor in MAIP1-mediated esophageal endothelial cell carcinogenesis. Considering the complex information in the dataset (specific details of the patient's drug or surgical treatment, etc.) and the lack of clear characterization, the above inferences still require rigorous basic experimental validation. Future studies aimed at determining the exact mechanism of MAIP1 in mediating the recruitment of different immune cell populations toward tumor progression or other immune-related processes should be conducted.

The immune system plays a critical role in eliminating malignant cells from healthy individuals. But unfortunately, tumor cells escape through immune-mediated infiltration to block clearance by immune infiltrating cells. Because of the ability of T cells-associated antitumor immunity, checkpoint inhibition has become a common clinical application in cancer immunotherapy [8]. Many large clinical trials have shown that immune checkpoint blockade (ICB) therapy might benefit chemotherapy resistance patients in ESCC, even as an individualized agent in palliative treatment [21, 22]. A related study found that tumor microenvironment component activity and associated therapeutic strategies could provide insights into the development of combination therapies for immune checkpoint blockade [23]. Therefore, we evaluated both MAOP1 and immune checkpoints in terms of clinical applicability. The EC subgroup that significantly differentially expressed MAIP1 was closely associated with TNFRSF18, HHLA2, PDCD1LG2, LGALS9, TNFSF15, TNFRSF14, TNFSF18, and CD274, indicating that targeting MAIP1 may synergize the antitumor properties of EC immunotherapy.

Our study has several limitations. First, the study relied exclusively on available information from one open-access database and did not combine multiple databases for a more comprehensive validation. Second, the assessment of MAIP1 expression was based exclusively on the mRNA levels reported in the aforementioned databases, which do not reflect the levels of functional proteins. Third, this is a preliminary study based on web-based tool, whose findings deserve further validation. Studies suggest that protein activity may be influenced by posttranscriptional modifications and/or regulation of protein hydrolysis [24]. In conclusion, our study suggests that MAIP1 can affect TME. However, the exact mechanism of MAIP1 in regulating the recruitment of specific immune cell populations to tumors or other immune-related processes remains unclear [25]. The direct role of MAIP1 and its contribution to EC progression needs to be verified experimentally. Although this study has some limitations, it provides clues to investigate the potential mechanisms of MAIP1 in EC and offers possible immunotherapeutic options and potential prognostic markers for EC.

## 5. Conclusion

In conclusion, we elucidated that MAIP1 is overexpressed in EC and intimately related to poor prognosis of EC. Besides, our results also confirmed that MAIP1 may promote the progression of esophageal cancer by reducing tumor immune cell infiltration and blockading immune checkpoint expression levels. However, the biomarkers developed in our study are limited by public databases and require further validation.

## Data Availability

All data were downloaded from TCGA (https://portal.gdc.cancer.gov/).
